# Deep-learning-based AI for evaluating estimated nonperfusion areas requiring further examination in ultra-widefield fundus images

**DOI:** 10.1038/s41598-022-25894-9

**Published:** 2022-12-17

**Authors:** Satoru Inoda, Hidenori Takahashi, Hitoshi Yamagata, Yoichiro Hisadome, Yusuke Kondo, Hironobu Tampo, Shinichi Sakamoto, Yusaku Katada, Toshihide Kurihara, Hidetoshi Kawashima, Yasuo Yanagi

**Affiliations:** 1 Department of Ophthalmology, Jichi Medical University, 3311-1 Yakushiji, Shimotsuke-Shi, Tochigi 329-0498 Japan; 2 DeepEyeVision, Inc, Jichi Medical University, Shimotsuke-Shi, Tochigi 329-0498 Japan; 3 Department of Ophthalmology, Keio University, Tokyo, 160-8582 Japan

**Keywords:** Retinal diseases, Machine learning

## Abstract

We herein propose a PraNet-based deep-learning model for estimating the size of non-perfusion area (NPA) in pseudo-color fundus photos from an ultra-wide-field (UWF) image. We trained the model with focal loss and weighted binary cross-entropy loss to deal with the class-imbalanced dataset, and optimized hyperparameters in order to minimize validation loss. As expected, the resultant PraNet-based deep-learning model outperformed previously published methods. For verification, we used UWF fundus images with NPA and used Bland–Altman plots to compare estimated NPA with the ground truth in FA, which demonstrated that bias between the eNPA and ground truth was smaller than 10% of the confidence limits zone and that the number of outliers was less than 10% of observed paired images. The accuracy of the model was also tested on an external dataset from another institution, which confirmed the generalization of the model. For validation, we employed a contingency table for ROC analysis to judge the sensitivity and specificity of the estimated-NPA (eNPA). The results demonstrated that the sensitivity and specificity ranged from 83.3–87.0% and 79.3–85.7%, respectively. In conclusion, we developed an AI model capable of estimating NPA size from only an UWF image without angiography using PraNet-based deep learning. This is a potentially useful tool in monitoring eyes with ischemic retinal diseases.

## Introduction

Fundus fluorescein angiography (FA) has been essential for diagnosis and precise monitoring of ischemic retinal diseases like diabetic retinopathy (DR) or retinal vein occlusion (RVO) because it is the only procedure that can delineate several important pathognomonic factors. For instance, FA uses dynamic images to show retinal microvascular change and to reveal features such as neovascularization (NV) by highlighting fluorescein dye^1,2^. As an indicator of severity, the nonperfusion area (NPA) is one of the most important features of microvascular injury seen during fundus FA of the eyes in ischemic retinal disease^3–5^. For example, the NPA has been the gold standard for differentiating between ischemic and nonischemic central retinal vein occlusion (CRVO), based on whether FA shows a 10-disc area of retinal capillary obliteration^6^. This differentiation is important because clinical features, complications, visual outcomes, and managements differ greatly between the two. However, although FA plays an important role in detecting pathological changes in retinal disease, it is time-consuming and there are potential side-effects such as nausea, vomiting, urticaria, vasovagal reaction, anaphylaxis, pyrexia, and death^7^. This makes FA unsuitable for frequent routine examinations. Generally, it is recommended when ophthalmologists or retinal specialists suspect a large NPA on clinical examination and need to look further for areas of ischemia. However, there is no consensus about when to conduct FA during monitoring of ischemic retinal disease.

Recently, ultra-widefield (UWF) imaging was developed for visualizing the retinal periphery^8^. UWF images have several advantages over conventional fundus photography. Of note, more than 80% of the retina can be captured on a single image, allowing detection and assessment of retinal lesions in wider areas than in conventional early treatment diabetic retinopathy study (ETDRS) seven-field fundus photography. With the more recent development of UWF fluorescent angiography (UWF-FA) imaging, studies have revealed distributions of NPA, NV, and other DR lesions in the posterior and peripheral retina in more detail^9–13^. These studies support the usefulness of UWF (and UWF-FA) imaging for detecting retinal lesions associated with ischemic retinal disease.

Deep learning, a form of machine learning using convolutional neural networks (CNN), offers unprecedented approaches to retinal imaging analysis. This form of artificial intelligence (AI) is the most common image analysis method, and increasingly many studies have reported potential applications to medical imaging, including retinal imaging modalities. Various studies have demonstrated automatic detection or segmentation of features like subretinal fluid in optical coherence tomography (OCT) ^14,15^ or choroidal neovascularization of OCT angiography^16^. Regarding FA modality, AI models can detect multi-label lesions of microaneurysms, NPA, and leakage in FA images^17^. Interestingly, in some area of medical imaging, AI can make predictions more precisely than can most specialists. Deep learning can even use color fundus photography to predict unobservable features, such as OCT-derived center-involved diabetic edema^18^.

We hypothesized that deep learning could be applied to estimate areas of significant NPA, such that the algorithm can use UWF retinal imaging to identify eyes at elevated risk of proliferative changes and thus requiring more detailed examination, such as FA. NPAs are characterized by its own coloration as well as by surrounding soft exudates, intraretinal microvascular abnormality, or vascular sheathing that continue upstream of the NPA^10^. Therefore, we used PraNet ^19^ as our network architecture, which was originally a polyp segmentation model that utilized PraNet’s reverse attention module. The reverse attention module estimates foreground regions in a coarse-to-fine manner, which is beneficial for nonsalient or camouflaged semantic segmentation like vascular sheathing. Conventional U-Net type network architectures those have no reverse attention modules recognize only the features of each region. Here, we propose a PraNet-based deep-learning model for estimating the size of NPA in pseudo-color fundus photos from an UWF imager (Optos California, Nikon, Tokyo), and evaluate the estimated NPA (eNPA; expressed as disk area (DA]) by comparing the size and the centroids of the eNPA with human-annotated regions as ground truth.

## Results

### Establishment of PraNet based model

We compared the performance of PraNet-based model to other network architecture which were used in previous reports, that is DeepLabv3, PSP Net and U-Net. Overall, the results demonstrated that PraNet outperformed other models. Although the bias was smallest with DeepLabv3 model (9.44mm^2^), followed by PraNet (− 31.8 mm^2^), PraNet performed best judging from the two most important parameters, i.e., confidence limits zone and centroid distance. (Supplemental Figs. 2, 3).

### Verification of eNPA

**Figure 1:**
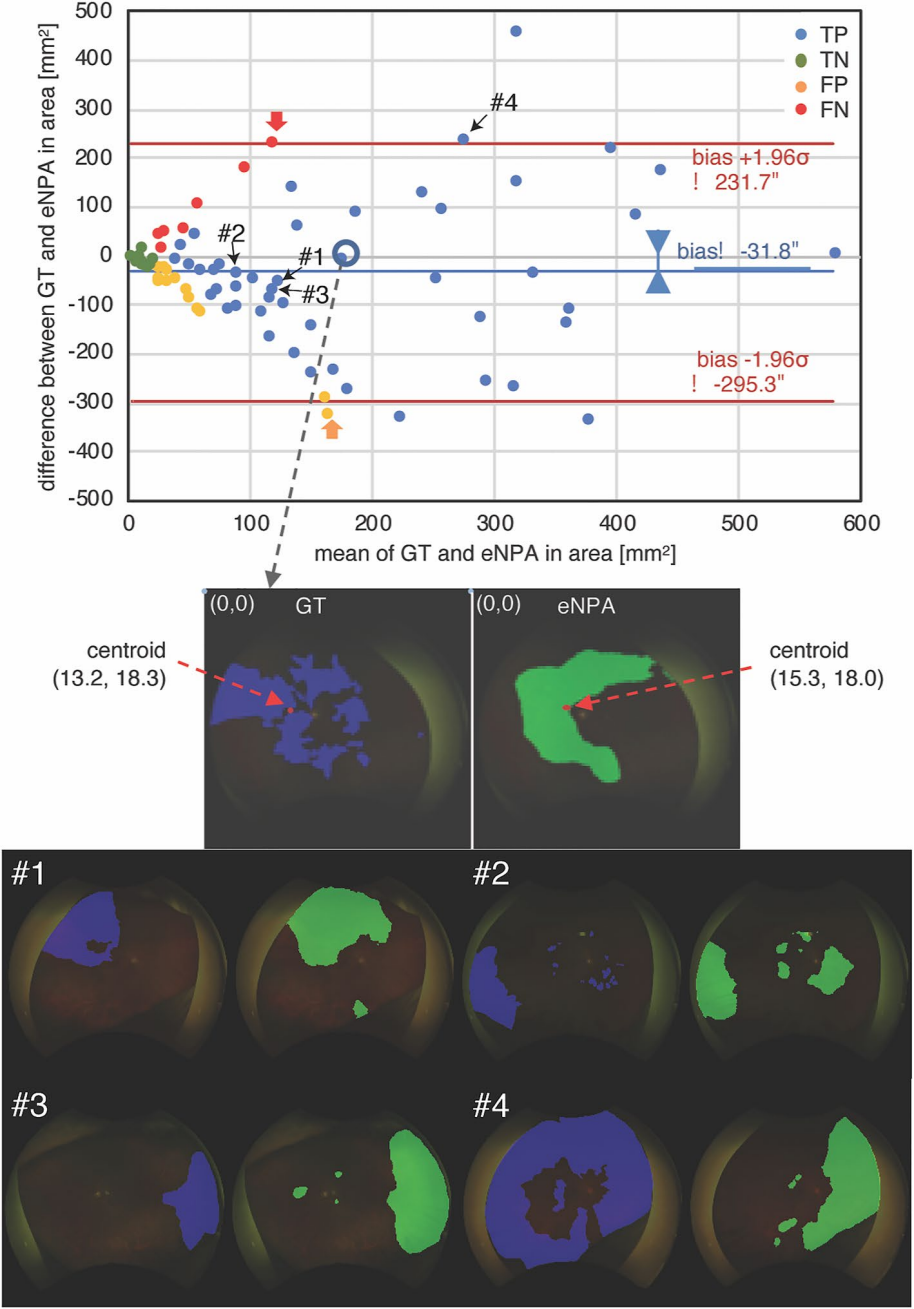
Bland–Altman plot (top) for verification and examples of ground truth/eNPA in the fine match case (bottom), including centroid locations. Bland–Altman analysis, a graphical scheme, drawing a scatterplot in which the X- and Y-axes represent the mean and difference of areas of the paired data (ground truth and eNPA), respectively. Point colors (difference between ground truth and eNPA) represent elements of the contingency table for validation, including true and false positives and negatives. The X-axis shows the mean area of the paired data (ground truth and eNPA), and point colors (centroid distance) represent elements of the contingency table, as in a Bland–Altman plot. The circled point corresponds to the fine match case. Limits of agreement are shown as the bias (solid blue line) with 1.96σ (solid red lines). Blue area in the bottom left fundus photo is the NPA ground truth, annotated using fluorescein angiography. Green area in the bottom right fundus photo is the AI-eNPA. #1: A poor quality image with eyelids. #2–4: Poor quality images with eyelashes.

Figure 1 shows a Bland–Altman plot (top) and one example of ground truth and eNPA, including locations of centroids in the fine match case (bottom). Both bias and the number of outliers met our goals; that is, the bias (− 31.8 mm^2^) was smaller than 10% of the confidence limits zone (52.7; = 10% of 2 × 1.96 × 134.4 mm^2^) and the number of outliers (6/80; 7.5%) was fewer than 10% (8) of observed paired images. Four of the 6 outliers were true positives. Therefore, in terms of clinical validity, we believe that eNPA by our AI model agrees well with delineation by experienced graders. Additionally, effects of eyelids or eyelashes were, which potentially affects the detection of peripheral lesions on UWF imaging, were investigated on low-quality images. Four typical low-quality images with eyelids or eyelashes are shown in the Bland–Altman plot (#1–4). The performance of the model on these images was not deemed poor, and the model successfully segmented e-NPA avoiding the artifacts from eyelids and eyelashes.

**Figure 2:**
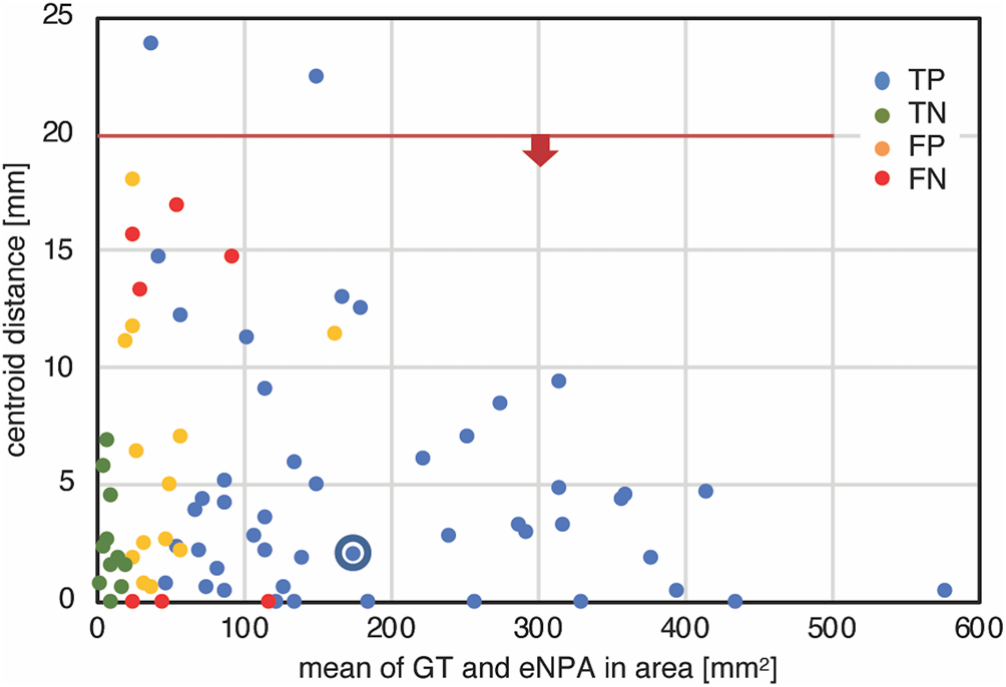
Plot of centroid distances. Limit of agreement is shown as 20 mm, which is approximately half the diameter of a flattened retina.

Figure 2 shows a distribution of 80 distances between GT and eNPA centroids. Here, the two outliers exceeding 20 mm (half the UWF diameter) is within the goal, and both are true positives.

### Inter-observer variation of expert annotation

**Figure 3:**
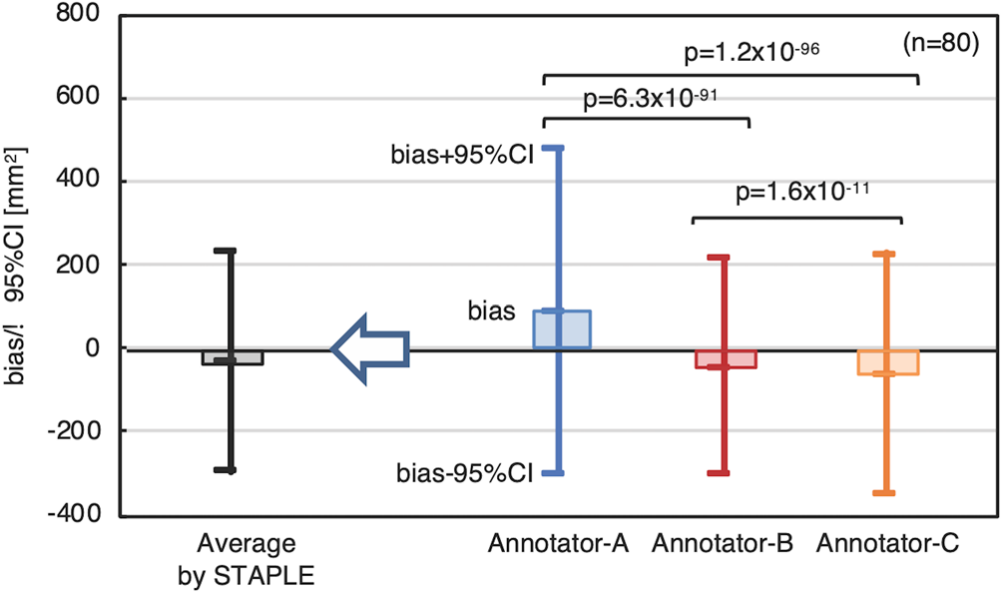
Bias and 95% CI for interobserver variation. Annotator B was the main annotator and closest to the eNPA.

**Figure 4:**
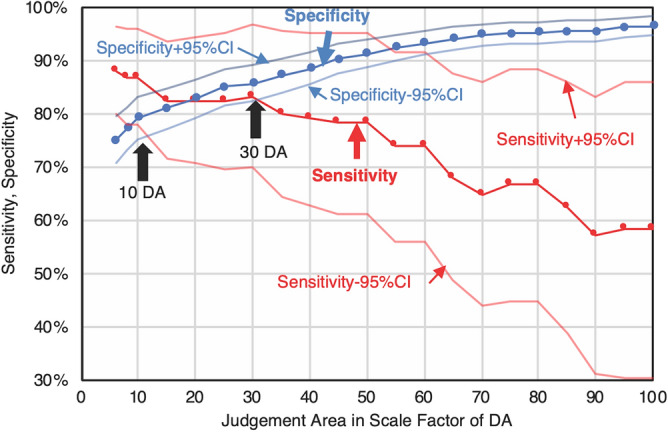
Influence of JA on the sensitivity and specificity in error analysis. Influence on the results of error analysis of changing JA using the contingency table (Table 2). Previous studies using normal-field fundus cameras suggested that 10 DA is the threshold of NV occurrence, but more recent studies using UWF suggest that 30 DA, or even 50 DA, is the threshold. Here, sensitivity and specificity remained at almost 80% until around 30 DA.

We analyzed inter-observer variation of annotations by three annotators according to bias and the 95% CI in a Bland–Altman plot (Fig. 3) using t-test statistical analysis between each annotator pair, Annotators A and B, Annotators B and C, and Annotators C and A. Figure 4 shows that averaging by STAPLE indicates that bias became low by balancing out biases among the three annotators and that the 95% CI range was kept small, as in the case of Annotator B.

### External validation study of eNPA

Supplemental Fig. 1 shows a Bland–Altman plot for external data. Although the model tended to over-estimate the eNPA, the bias (− 34.6 mm^2^) was almost same as the result of our verification using an internal dataset (− 31.8 mm^2^) and the number of outliers (9/133; 6.7%) was fewer than the result.

### Influence of judgment area on the results of error analysis

Next, we investigated the influence of JA on the results of error analysis. Figure 4 shows the influence of JA on sensitivity and specificity in this study. At 10 DA, the sensitivity and specificity were 87.0% (95% CI 78.1 to 96.0) and 79.3% (95% CI 75.3 to 83.3), respectively. The best balance between sensitivity and specificity was at 30 DA, with 83.3% (95% CI 70.0 to 96.7) sensitivity and 85.7% (95% CI 82.4 to 89.1) specificity.

## Discussion

We demonstrated an AI model for evaluating eNPA judged as requiring more detailed examination. The AI had sensitivity and specificity values of 0.870 and 0.793 using 370 UWF images of nonischemic fundus and 80 UWF images of various ischemic retinal diseases, DR, RVO, and posterior uveitis. AI models described in previous reports could segment retinal characteristic findings like microaneurysms, NPA, and leakage in FA images of a single specific disease. To the best of our knowledge, the developed AI is the first capable of evaluating eNPAs in fundus photographs and alerting physicians to consider performing further detailed invasive examinations. An UWF image scan can be obtained non-invasively. This is especially beneficial when following up people who have had a mild allergy after an angiography and those with liver or kidney disfunction.

Ischemic retinal diseases are characterized by structural and functional alteration in the retinal microvasculature, which in turn causes microvascular occlusion, hemorrhage, microaneurysm, venous beading and intraretinal microvascular abnormality. These microvasculature ultimately results in NPA and NV. Major segmentation AI in the medical field, such as U-Net and DeepLabv3, learns segmentation lines that separate regions with different patterns, and is not suitable for the task required in this study, which is to determine that the region is roughly this region by referring to findings associated with surrounding ischemia and blood vessel runs in a wide surrounding area, instead of separating the region based on image patterns alone. We chose PraNet because it is a medical image segmentation model that has attention module^19^. Attention module let the model to exploit non-neighboring area. For accurate segmentation of NPA, the model should focus on not only NPA itself but on its surrounding region.

There are several reports investigating the possible use of color fundus photograph to generate parameters obtained from FA. For example, a previous report showed that a deep learning model produced high quality FA images that were indistinguishable from real angiograms^20^. In this report, they used fundus images to generate FA images. This report is similar to the current study in that it used deep learning to estimate fundus images from one modality to another. Although this report is also superior in that it produced a very clear FA image that showed vascular structures, they did not evaluate estimation of extravascular NPAs. Other previous reports which aimed to identify NPAs from FA images used other network architecture instead of PraNet^17,21^. These studies were similar to the current study in that they estimated NPAs using with CNN, but they differed from our studies in that they estimated NPAs from FA images instead of color images.

The developed system has some advantages over OCT-A. Now we can obtain high-resolution images of retinal and choroidal microvasculature using with OCT-A. As fundus imaging technology has developed, so have technologies for wide-angle imaging in OCT-A. Recently, some studies have investigated widefield swept-source OCT-A (WF-OCT-A) as an alternative to UWF-FA for surveying ischemic retinal diseases like DR and RVO^22–25^. While this may prove to be a useful noninvasive tool, there are some difficult-to-resolve barriers to actual clinical use. As pointed out by Spaide et al.^26^, the eyes must fixate for a certain period of time to obtain clinically useful WF-OCT-A images, which may be difficult for some patients. For example, patients with diabetes may have difficulty fixating due to macular edema. Second, although WF-OCT-A offers a wider field of view than OCT-A, it is still smaller than UWF images. Especially in cases of surveying proliferative diabetic retinopathy (PDR), peripheral ischemia is an important factor in proliferative changes^4,8^. Last but not least, a WF-OCT-A instrument is more expensive than an UWF instrument.

In this study, the AI defined the area for labeling as NPA in the range of confidence 0.5 or greater. Supplemental Fig. 4 shows a color map of how an AI program estimates NPA. Decreasing the threshold, which improves sensitivity but detoriorates specificity, widens the estimated NPA. The figure shows how the area of eNPA changes depending on the confidence level.

In this study, we followed convention by adopting 10 DA as the judgement area^6^. However, some studies have shown that in practice 10 DA of NPA on FA is an unreliable parameter for differentiating between ischemic and non-ischemic CRVO^4,27^. Moreover, there are reports that in various ischemic retinal diseases, retinal capillary nonperfusion starts first at the peripheral retina and then progresses toward the posterior pole. The development of UWF-FA now allows imaging of large areas of the retina in a single image, which has shown that higher DR severity has a higher association with a larger area of NPA and NV than was previously thought^8,28–30^. Studies of PDR progression have shown that NPA of about 77.48 mm^2^ (almost 31 DA) to 107 DA indicate increased risk of developing PDR^4,27^.

Figure 4 shows that the developed AI model can achieve over 80% for both specificity and sensitivity in eyes with NPA of 10–35 DA. Although the sensitivity decreased to under 60% in an eye with a large NPA, around 100 DA, the developed AI model shows not only recommendation levels for further examination like FA, but also the eNPA. A Bland–Altman plot based on both internal and external validation showed that there was little difference between the eNPA and ground truth (Fig. 1, Supplemental Fig. 1), suggesting that the eNPA will be large when the ground truth is large. We attribute the decrease of sensitivity in large NPAs to the design of the loss function. To detect small NPA regions with detailed boundaries, the loss function is calculated with a large weight on boundaries. Therefore, when the NPA region is large, the loss function puts small weight on misclassification inside the NPA region. Either way, physicians can then decide the need for further examinations based on their own ophthalmoscopic examinations, other image modalities, estimated NV presence, or NPA if it was large.

The advantage of the proposed method is its high versatility. The training dataset did not exclude images like eyes with post-photocoagulation, vitreous hemorrhage, or poor resolution due to cataracts. Moreover, we included images of a variety of retinal ischemic diseases including DR, RVO, ocular ischemic syndrome, and uveitis. We believe our results demonstrated that a properly trained AI with minimal exclusion of poor images lead to the AI which is able to be used in real-world.

This study has some limitations. This study did not evaluate models other than Optos California. Furthermore, specificity was relatively low—0.793 (0.753–0.832)—instead of high specificity. However, if the AI estimates NPA in the eyes of a person without disease, the ophthalmologist can dismiss the need for an angiography based on the history and fundus findings. Third, the without-NPA images included those that have not undergone FA. Although in the current study ages or sex were not significantly different between images with NPA and without NPA, NPAs are present in peripheral retinal areas even in healthy subjects especially those who are older. Finally, a 95% CI of specificity is clearly much narrower than that of sensitivity due to a sufficient amount of non-NPA data (370 images without NPA vs 80 images with NPA). Larger real-world studies are needed to confirm our findings.

We developed an AI model that can use only UWF images to make recommendations for ophthalmologists about the need for further detailed examinations such as FA. PraNet architecture, which recognize features characterized by surrounding soft exudates and/or microvascular abnormality, well estimated NPAs. The proposed method for evaluating NPA without angiography with UWF, which does not require mydriasis, will reduce the burden on patients and ophthalmologists and reduce unnecessary examinations while increasing necessary examinations.

## Methods

### Study design and approval

The institutional review board of Jichi Medical University (Jichi-CU19-094) approved this retrospective study at 2 centers. The study procedures adhered to the tenets of the Declaration of Helsinki and followed institutional guidelines, and we obtained informed consent in the form of an opt-out on the Jichi Medical University Department of Ophthalmology website. Where necessary, all patients provided informed consent to procedures performed as part of their clinical management.

### Subjects

The training NPA images were 1725 patients of 17,600 images, those of mean age was 59.6 years and insisted of 1000 males. The training without NPA were 3438 patients of 16,000 images, those of mean age was 59.0 year and insisted of 1852 males. When we extracted the images from the electronic medical records, we did not select the images by image quality. Therefore, the quality of the dataset is on the same level as real-world data. The FA images were annotated by a well-trained grader who had underwent multiple training sessions for the current segmentation for NPA. The annotations were double-checked and curated by a senior retinal specialist.

### AI modeling

Primary output of the current system was the area size and centroid of the eNPA, which is a judgment measure when performing retinal laser coagulation or FA imaging. Since the limited sizes of receptive fields has made it difficult for conventional CNNs to predict NPA, we incorporated an attention mechanism into CNNs as a viable approach to widening receptive fields without overly increasing computational costs. In brief, for accurate segmentation of NPA, the predicting model should focus not only on the NPA itself but also on its surrounding region, since a noncontiguous area is a helpful clue for estimating NPA in a neighborhood. For instance, the NPA distribution correlates with that of vascular sheathing, but conventional CNNs are too shortsighted to capture meaningful features from peripheral pixels. An attention mechanism allows CNNs to consider noncontiguous areas, presumably improving estimation accuracy.

We used PraNet ^19^ network architecture that utilized PraNet’s reverse attention module. We hypothesized that the reverse attention module is an effective cue in NPA segmentation. We verified our hypothesis by comparing PraNet and DeepLabv3, which is a modern network architecture of the well-known U-Net type network architectures and does not have attention module. In our preliminary examination, PraNet achieved about 10 points higher Dice score than DeepLabv3 on our validation set.

**Figure 5:**
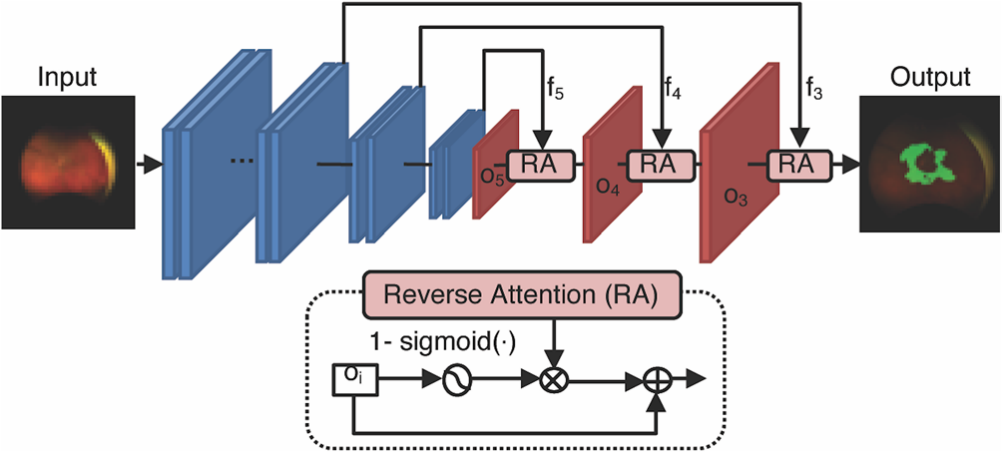
PraNet Diagram.

We used a pretrained weights available at PraNet’s website (https://github.com/DengPingFan/PraNet). We combined focal loss ^31^ and weighted binary cross-entropy loss ^32^ to speed up the convergence of training loss and to lessen complexity of the loss landscape (Fig. 5). Focal loss is designed to address class imbalance in the training dataset by adjusting the loss decay in each class. Supplemental Table 1 shows the data splitting in our experiment, which is highly imbalanced; the number of without-NPA images is five times larger than NPA ones. Focal loss weight is more on the small-number class (NPA) and less on the large-number class (without-NPA), avoiding over-fitting on the large-number class. Weighted binary cross-entropy loss more heavily penalizes wrong estimations of the segmentation boundary area. We expect structure loss to enforce the model to focus on the informative peripheral NPA area.

To mitigate overfitting due to the small number of training images, we applied several data augmentations. We applied the following data augmentation techniques in training; random crop and random affine transformation, such as translation, flipping, random resize, and rotation. We believe that the shape of the vessels is critical in determining NPA, and that color, contrast, and brightness are nonessential properties. As in the general machine learning pipeline, data augmentation is performed only during training phase. Specifically, we randomly changed the brightness, contrast, aspect ratio, scale, and saturation of input images. We used Adam ^33^ as the optimizer. We set the learning rate to 2.0 × 10^–5^ and the weight decay to 1.0 × 10^–5^. We decayed the learning rate by a factor of 3 every 10 epochs. To reduce memory consumption, we reduced the image resolution from 4000 × 4000 to 2000 × 2000. We trained the model over 120 epochs and measured the test accuracy with the best model at the validation phase. To increase the training data, we randomly sampled 400 without-NPA images in each training epoch. For hyperparameter optimization of the above data augmentations and optimizer strategy, we used Optuna^34^, an automatic hyperparameter optimization software framework particularly trained for machine learning. Optuna searched for the best data augmentation and learning schedule to minimize validation loss. We explored hyperparameters in 30 trials, which required 5 days of training using 8 Tesla-V100 GPUs. Inference of each image using this model took about 48 s. For real-world applications, we quantized the model for Intel CPU processors with OpenVINO. ^35^ The optimized model took only about 7 s for inference without significantly worsening its Sørensen–Dice coefficient (36.6 and 35.3, before and after optimization). This code is publicly available at https://github.com/DeepEyeVision/pred-npa-on-ufw.

### Verification

To verify our AI model for NPA estimation, we used 80 paired images of a color fundus image (UWF image) and its FA image (UWF-FA image), acquired using an UWF fundus camera (Optos California, Nikon, Tokyo). The paired images included DR, RVO, and other ischemic retinal diseases. Two experienced retinal specialists (H.T. and Y.Y.) confirmed the presence of NPA in the UWF-FA images. As shown in Supplemental Fig. 5, a trained orthoptist first manually aligned the magnification ratio, shift, and rotation of the UWF and UWF-FA images by using an AI development platform (Menou-TE, Menou, Tokyo) to superimpose them. Supplemental Fig. 6 summarizes the procedure for creating ground truth for verification. Three graders independently segmented the NPA with closed boundaries on the UWF-FA image, which the platform automatically aligned with the UWF image. One or more NPA annotations were generated in each UWF. We applied positioning information describing adjustments for 80 UWF-FA and UWF image pairs to each annotation session. We used the simultaneous truth and performance level estimation (STAPLE) method^36^, a widely used algorithm for obtaining a standard reference with statistical fusion, to combine annotated NPA regions as the ground truth. We used bias and 95% confidence intervals in a Bland–Altman plot to evaluate inter-observer variation of annotations.

As the characteristics of UWF like Optos, the images sometimes include artifacts such as eye lashes. We avoided to annotate such artifacts like peripheral black area during annotation.

### Statistical analysis for verification

For comparative verification of areas, we used Bland–Altman analysis ^37^ to quantify agreement between the ground truth and eNPA. Namely, we quantified the bias as systemic error and its 95% confidence limits (bias ± 1.96σ, where σ is the standard deviation of areas of paired data). Our goals for these two parameters were for bias to be within 10% of the confidence limits zone (2 × 1.96σ) and for the number of outliers beyond the limits to be within 10% of the number of observed paired images.

Assuming that accurate similarity measures between the ground truth and eNPA are not necessary for the current system, we used the distance between the centers of gravity of the ground truth and the eNPA, the “centroid distance” with intersection over Union (a same metric of Jaccard similarity index), rather than the Sørensen–Dice coefficient (another measure of similarity), to confirm the level of concordance. The specification of centroid distance is not particularly relevant in clinical terms, but we arranged the conditions such that the number of outliers beyond a half diameter of the fundus image is within 5% of the number of paired images.

### Validation

In the external validation study, we used 80 fundus images with NPAs and 370 control images exhibiting a variety of diseases such as cataracts and age-related macular degeneration but without NPAs. Those data set were taken in a clinic. We used a two-by-two contingency table to perform error analysis of judgments in laser coagulation treatments by means of a NPA threshold value for the area. Reportedly, retinal neovascularization will develop in one-third of eyes with central retinal vein occlusion when the NPA is larger than 10 DA, where 1 DA is the area of the optic disk, approximately 2.5 mm^2^ in images by a conventional fundus camera^6^. We initially set the judgement area (JA) as 10 DA. Table 1 shows the contingency table for error analysis in terms of JA in each ground truth–eNPA pair. We also investigated the influence of change in JA on the results of error analysis using the contingency table.

**Table 1:** Contingency table for validation.

	GT > *JA*	GT ≤ *JA*
eNPA > *JA*	TP: true positive (sensitivity)	FP: false positive
eNPA ≤ *JA*	FN: false negative	TN: true negative (specificity)

*GT* ground truth, *JA* judgement area, *NPA* non-perfusion area.

We specified that goals for sensitivity and specificity in clinical validity could be configured in FA imaging, considering the seriousness of a false positive, where the patient risks severe side effects due to the contrast media for FA imaging, or a false negative, which risks blindness. Table 2 shows our goals for sensitivity and specificity.

**Table 2:** Goal of sensitivity and specificity in estimated NPA.

Assumed goal	Sensitivity	Specificity
Judgement of FA imaging	> 80%	> 70%
Assessment of severe NPDR and PDR in DR	NPA less than 10 DA has a low risk of blindness and can be overlooked by 20% as false negative	It is not necessary to have a majority because FA imaging is not carried out other than for relevant diseases

*NPA* non-perfusion area, *FA* fluorescein angiography, *NPDR* non-proliferative diabetic retinopathy, *DA* disk area.

## Supplementary Information

Supplementary Figure S1.

Supplementary Figure S2.

Supplementary Figure S3.

Supplementary Figure S4.

Supplementary Figure S5.

Supplementary Figure S6.

Supplementary Table S1.

## Data Availability

The datasets used and/or analyzed during the current study are available from the corresponding author on reasonable request.
