# Supplementary material for: Deep-learning-based AI for evaluating estimated nonperfusion areas requiring further examination in ultra-widefield fundus images
Source: Sci Rep. 2022 Dec 17;12:21826. doi: 10.1038/s41598-022-25894-9 (PMC9759556; doi:10.1038/s41598-022-25894-9)
Supplement: Supplementary file 1 — Supplementary Figure S1. [file 41598_2022_25894_MOESM1_ESM.pdf]

Supplemental Figure 1 A Bland-Altman plot for external data of a clinic.

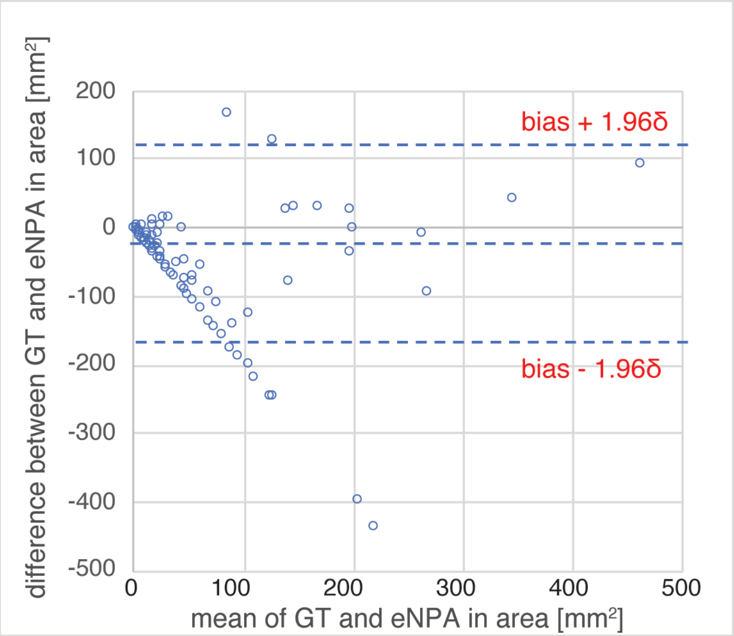

A Bland-Altman plot for external data of a clinic.

NPA was confirmed with fluorescein angiography in all eyes,

there was little difference between the estimated NPA and ground truth.

There are 32 plots at (0, 0).
