# Supplementary material for: Deep-learning-based AI for evaluating estimated nonperfusion areas requiring further examination in ultra-widefield fundus images
Source: Sci Rep. 2022 Dec 17;12:21826. doi: 10.1038/s41598-022-25894-9 (PMC9759556; doi:10.1038/s41598-022-25894-9)
Supplement: Supplementary file 2 — Supplementary Figure S2. [file 41598_2022_25894_MOESM2_ESM.pdf]

Supplemental Figure 2A A Bland-Altman plot using with DeepLabv3 model.

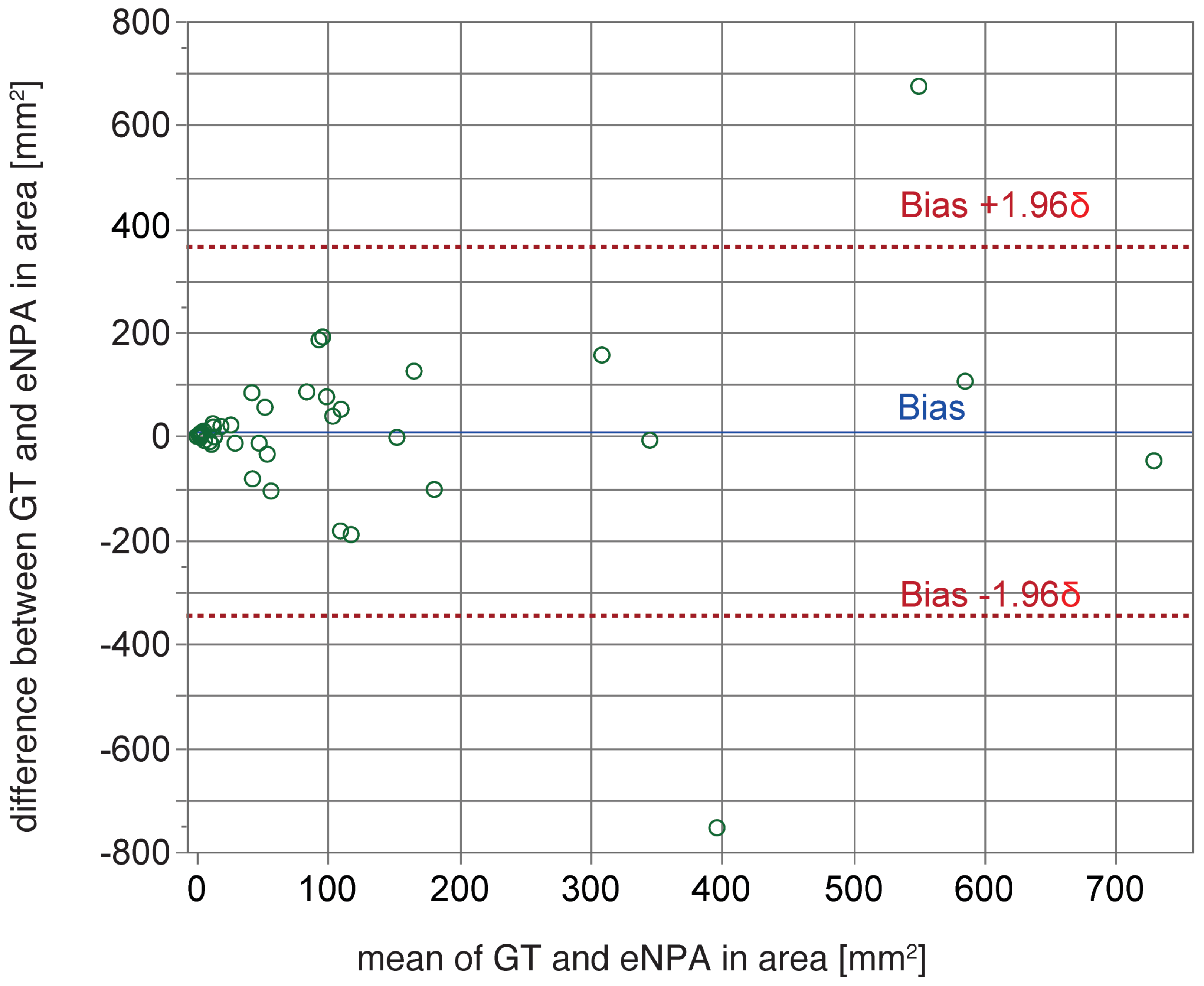

Supplemental Figure 2B A Bland-Altman plot using with PSPNet model.

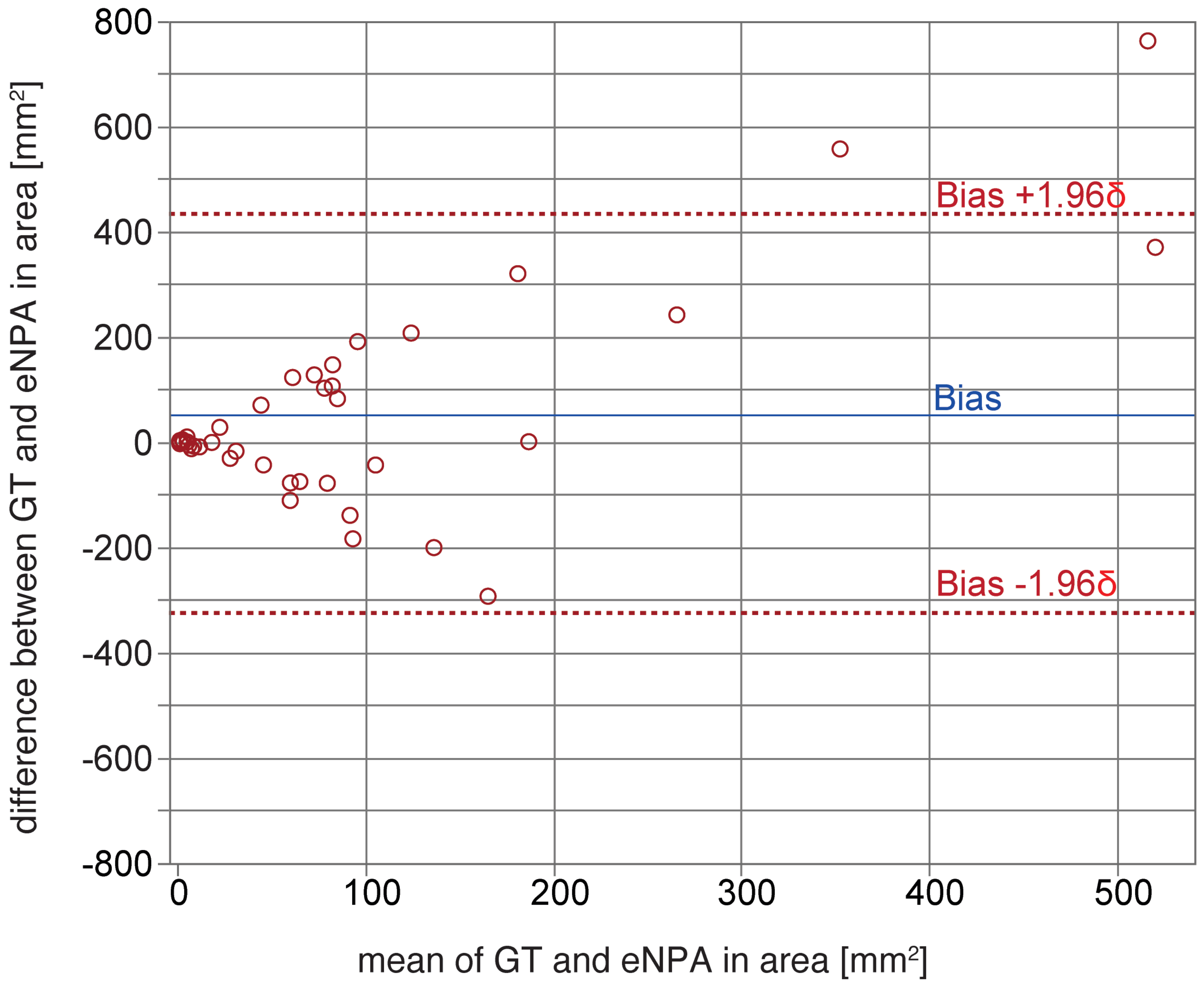

Supplemental Figure 2C A Bland-Altman plot using with U-Net model.

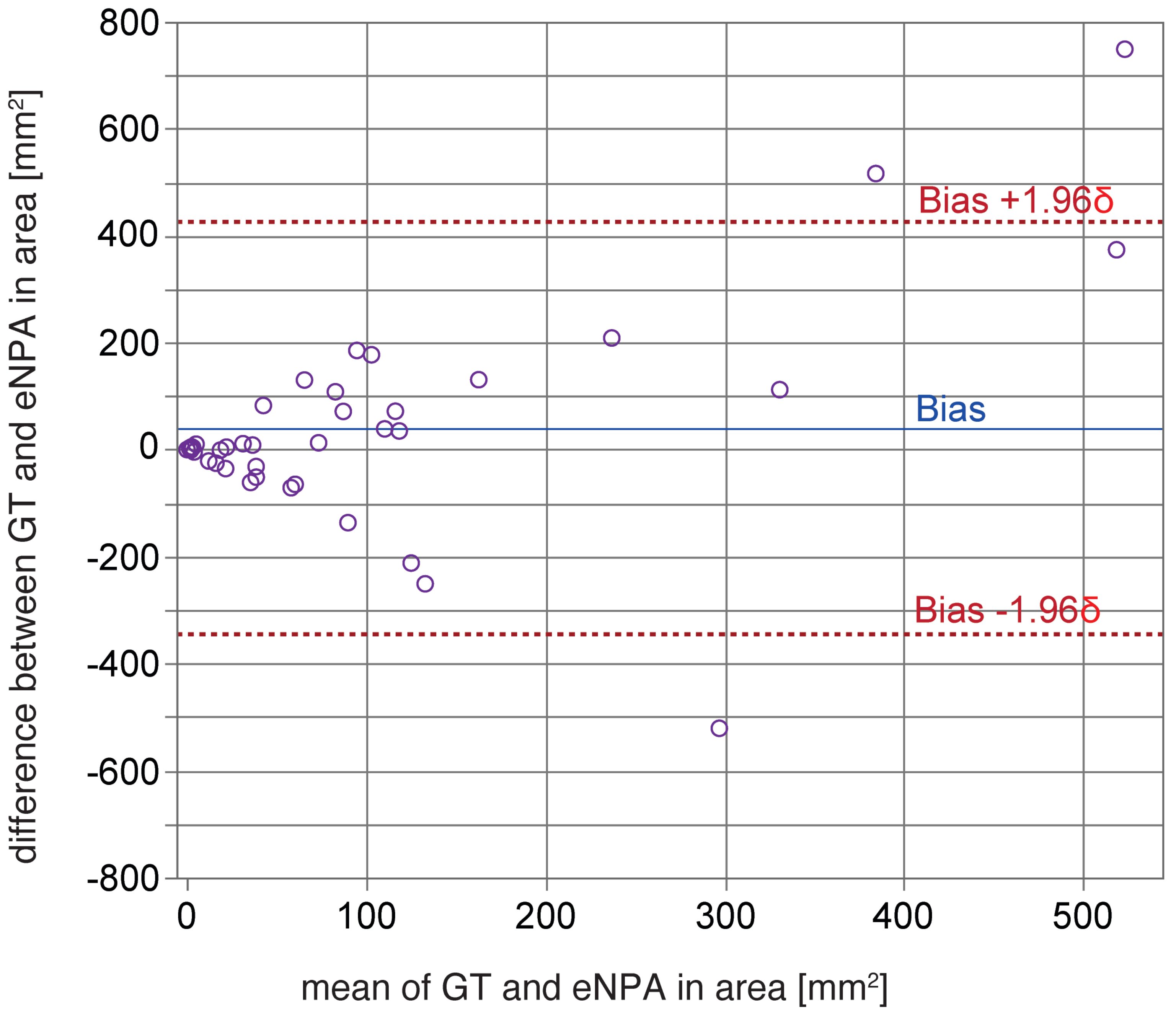

Bland-Altman plots shows the results of the association between GT and eNPA that estimated by three network architectures; DeepLabv3, PSPNet and U-net.

The bias between the eNPA and GT estimated by DeepLabv3, PSPNet and U-net (standard deviation) was 9.44 (180.7), 53.1 (193.4) and 39.0 (196.8), respectively.
