# Supplementary material for: Deep-learning-based AI for evaluating estimated nonperfusion areas requiring further examination in ultra-widefield fundus images
Source: Sci Rep. 2022 Dec 17;12:21826. doi: 10.1038/s41598-022-25894-9 (PMC9759556; doi:10.1038/s41598-022-25894-9)
Supplement: Supplementary file 3 — Supplementary Figure S3. [file 41598_2022_25894_MOESM3_ESM.pdf]

Supplemental Figure 3A Plot of centroid distances using with DeepLabv3 model.

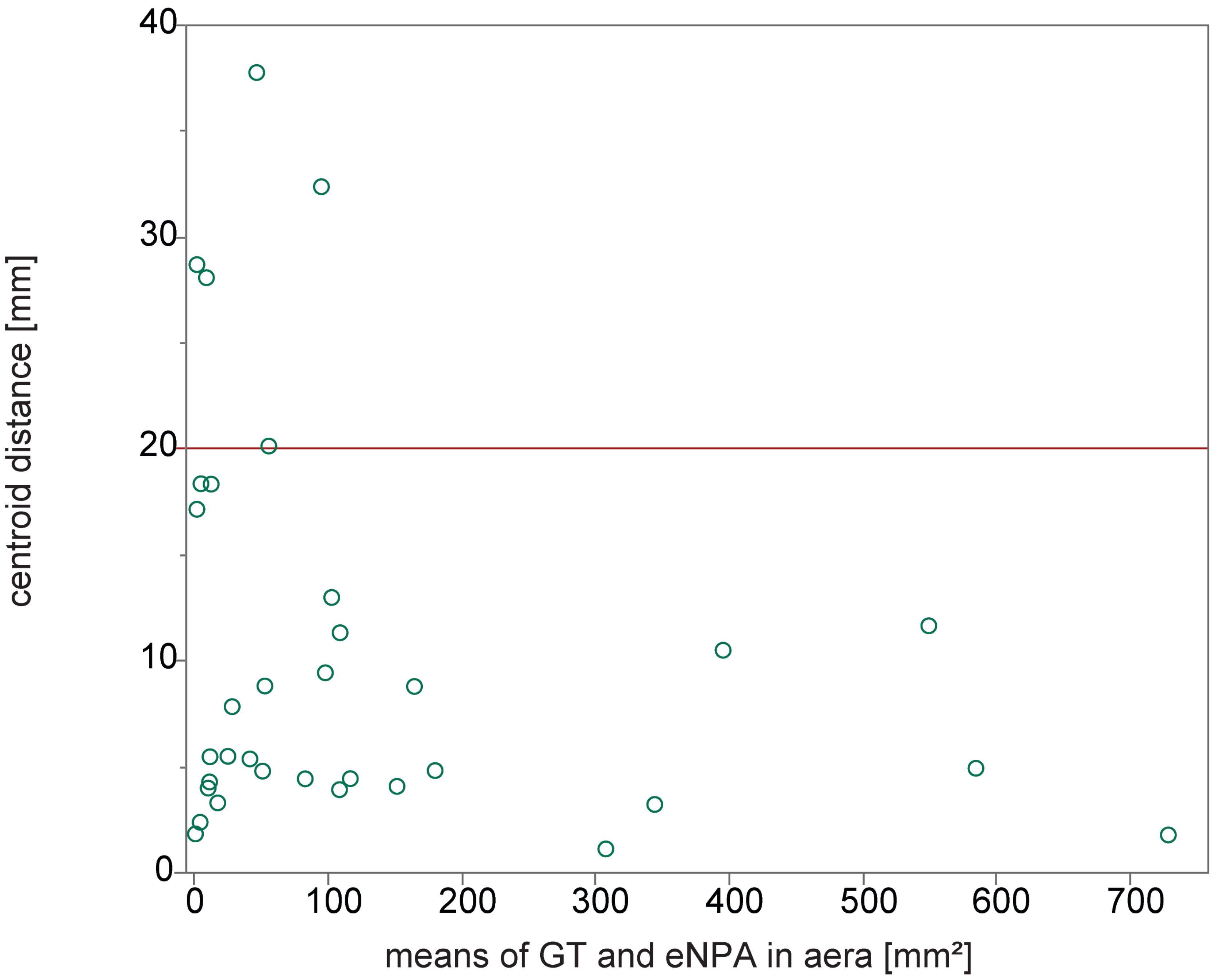

Supplemental Figure 3B Plot of centroid distances using with PSPNet model.

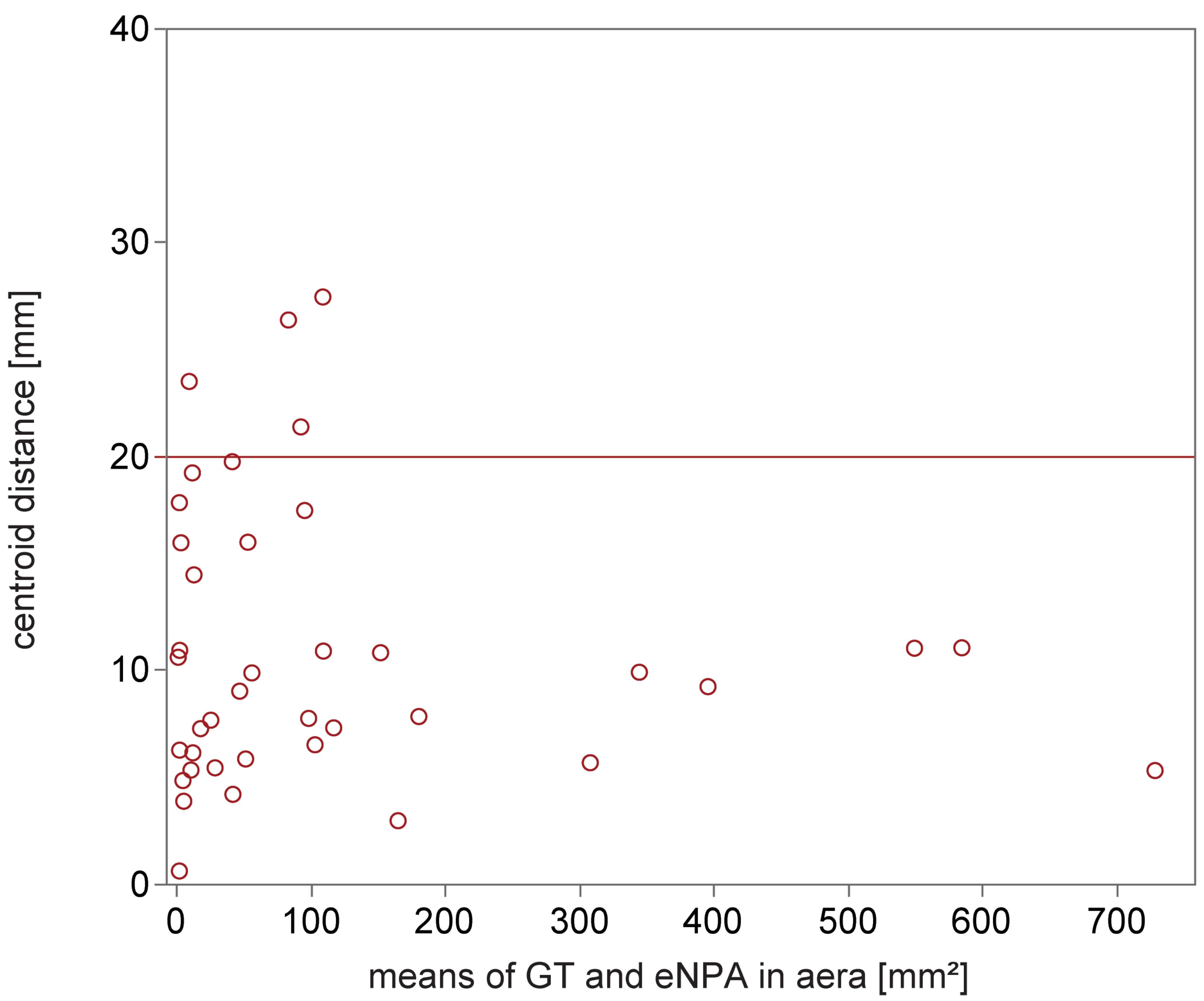

Supplemental Figure 3C Plot of centroid distances using with U-Net model.

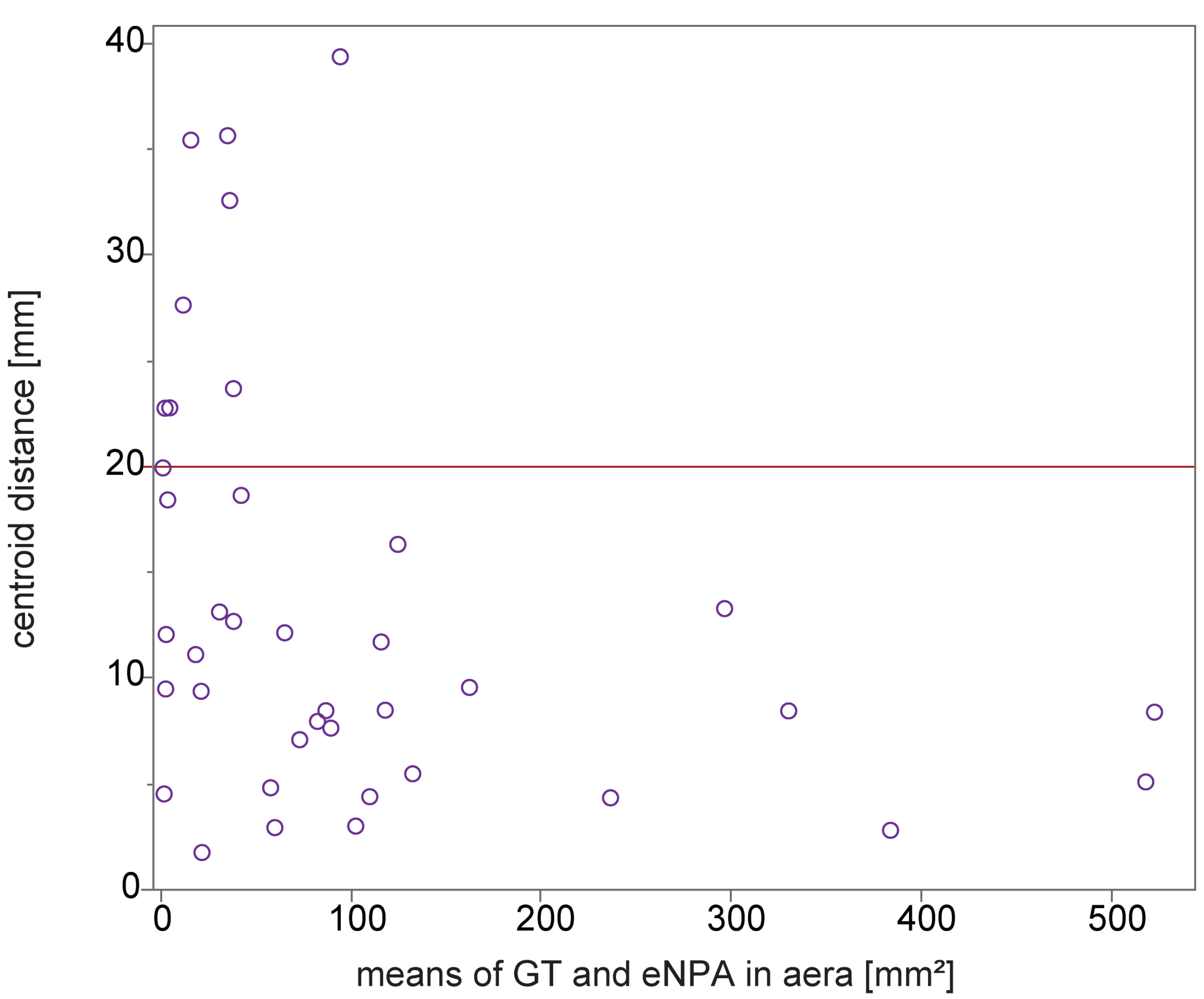

Plot of centroid distances shows a distribution of 40 distances between GT and eNPA centroids that estimated by three network architectures; DeepLabv3, PSPNet and U-net.

There are five, four and eight outliners exceeding 20 mm (half the UWF diameter) in the plot of centroid distances that estimated by DeepLabv3, PSPNet and U-net, respectivelye.
