# Supplementary material for: Deep-learning-based AI for evaluating estimated nonperfusion areas requiring further examination in ultra-widefield fundus images
Source: Sci Rep. 2022 Dec 17;12:21826. doi: 10.1038/s41598-022-25894-9 (PMC9759556; doi:10.1038/s41598-022-25894-9)
Supplement: Supplementary file 4 — Supplementary Figure S4. [file 41598_2022_25894_MOESM4_ESM.pdf]

Supplemental Figure 4 Ground truth (GT) and estimated-non perfusion area (eNPA) on the image.

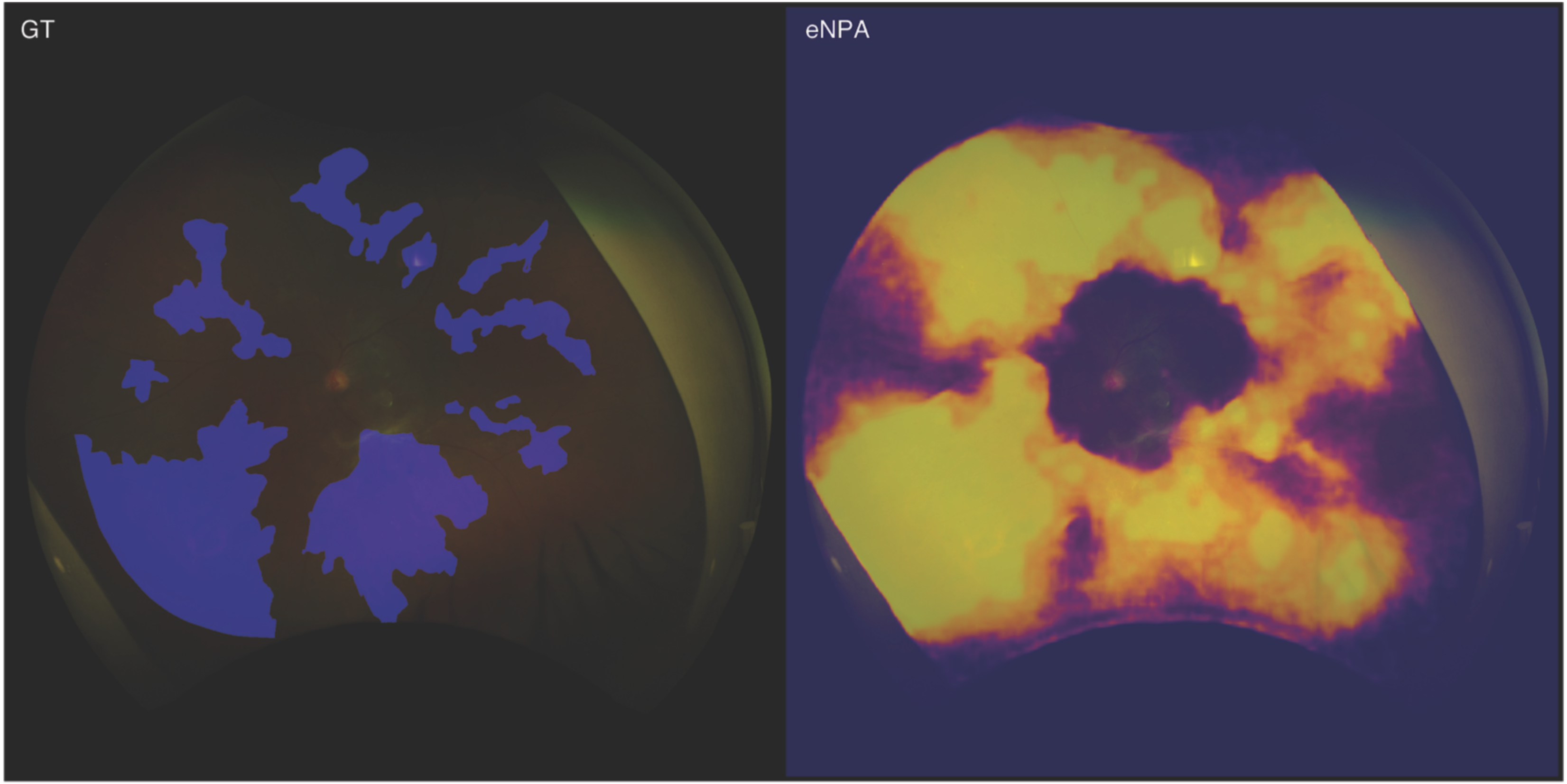

Blue area shows the ground truth and yellow area shows eNPA based on the confidence level.

More intense colors indicate higher confidence.
