# Supplementary material for: Deep-learning-based AI for evaluating estimated nonperfusion areas requiring further examination in ultra-widefield fundus images
Source: Sci Rep. 2022 Dec 17;12:21826. doi: 10.1038/s41598-022-25894-9 (PMC9759556; doi:10.1038/s41598-022-25894-9)
Supplement: Supplementary file 5 — Supplementary Figure S5. [file 41598_2022_25894_MOESM5_ESM.pdf]

Supplemental Figure 5 Annotated NPA region and its UWF-FA

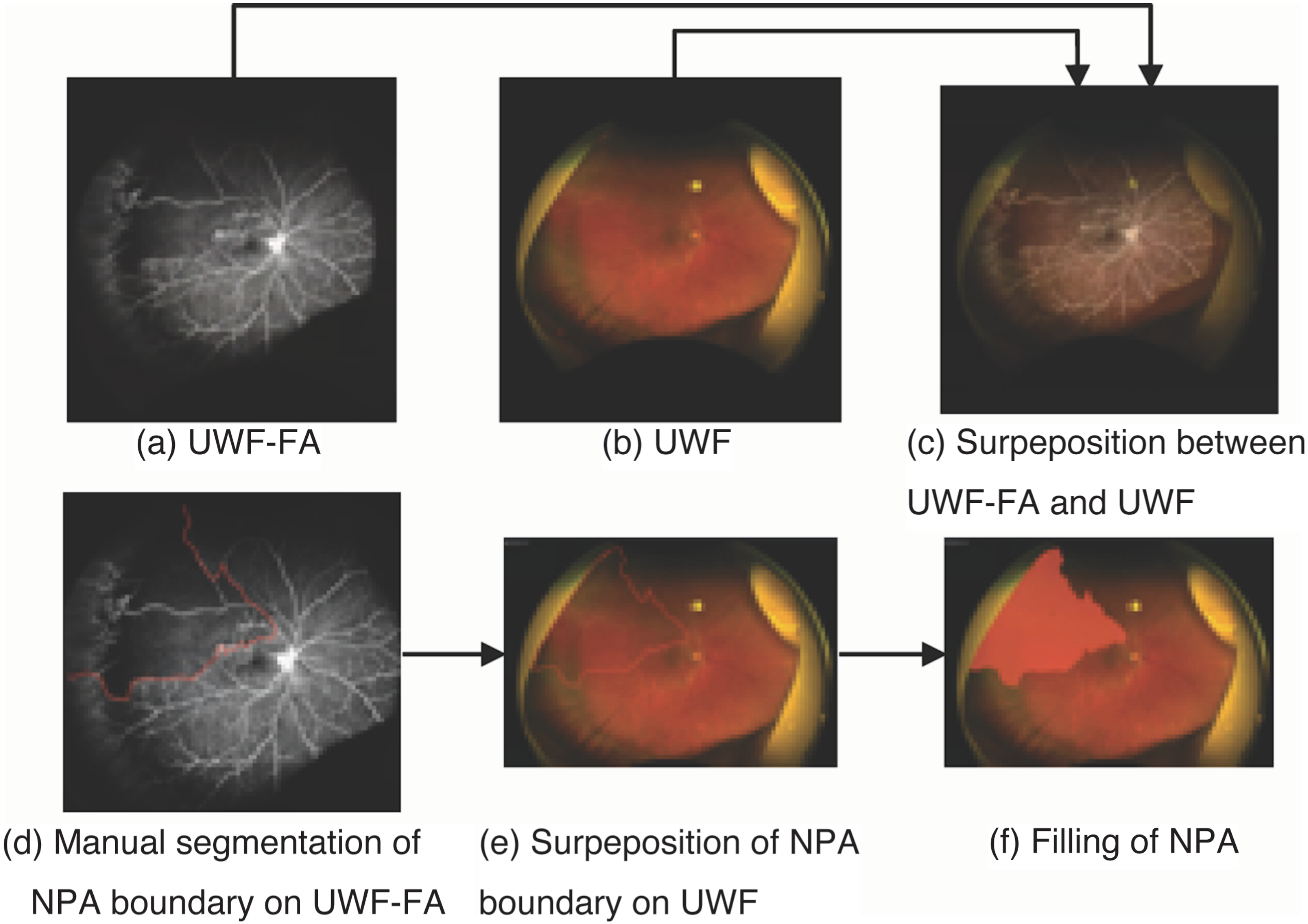

A trained orthoptist first manually aligned the magnification ratio, shift, and rotation of the UWF and UWF-FA images by using an AI development platform to superimpose them. We segmented NPA boundary on UWF-FA by manually, and supermposed them in UWF.
