# Supplementary material for: Deep-learning-based AI for evaluating estimated nonperfusion areas requiring further examination in ultra-widefield fundus images
Source: Sci Rep. 2022 Dec 17;12:21826. doi: 10.1038/s41598-022-25894-9 (PMC9759556; doi:10.1038/s41598-022-25894-9)
Supplement: Supplementary file 6 — Supplementary Figure S6. [file 41598_2022_25894_MOESM6_ESM.pdf]

Supplemental Figure 6 Procedures of verification

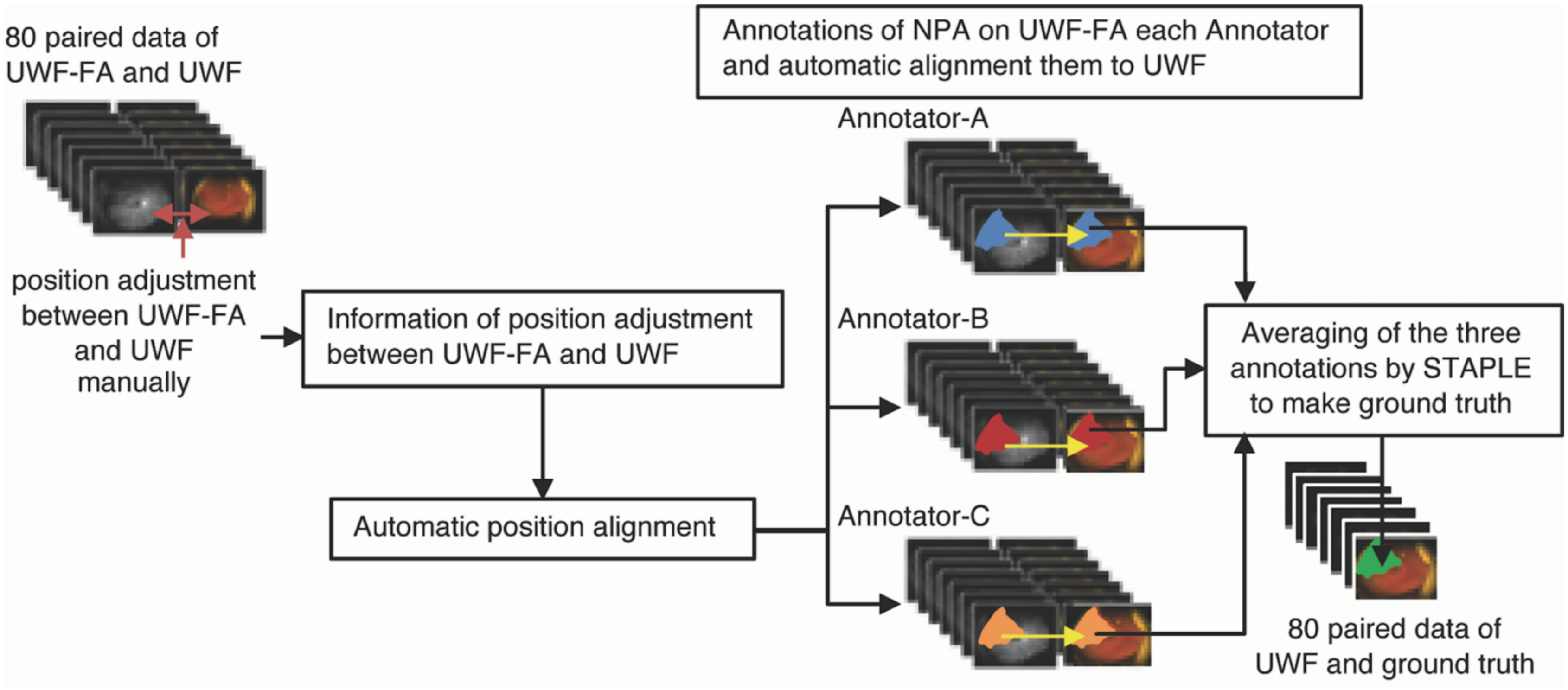

Two retina specialists and a photographer annotated to create the ground truth, but the photographer who created the training data performed all positioning of the color fundus photographs and the fluorescein angiography.
