# Supplementary material for: Deep-learning-based AI for evaluating estimated nonperfusion areas requiring further examination in ultra-widefield fundus images
Source: Sci Rep. 2022 Dec 17;12:21826. doi: 10.1038/s41598-022-25894-9 (PMC9759556; doi:10.1038/s41598-022-25894-9)
Supplement: Supplementary file 7 — Supplementary Table S1. [file 41598_2022_25894_MOESM7_ESM.pdf]

Supplementary Table 1. Data splitting

	Train	Val	Test
NPA	562	40	40
Non-NPA	2699	100	1000

Train, training data; Val, validation data; Test, test data; NPA, non-perfusion area.
